# Studying the Role of Novel Carbon Nano Tubes as a Therapeutic Agent to Treat Triple Negative Breast Cancer (TNBC) - an *In Vitro* and *In Vivo* Study

**DOI:** 10.30683/1929-2279.2024.13.06

**Published:** 2024-11-11

**Authors:** Kamal Asadipour, Narendra Banerjee, Jazmine Cuffee, Karrington Perry, Shennel Brown, Anasua Banerjee, Erik Armstrong, Stephen Beebe, Hirendra Banerjee

**Affiliations:** 1 Frank Reidy Research Center for Bioelectrics, Old Dominion University, Norfolk, VA-23529, USA; 2 Department of Natural Sciences, Pharmaceutical and Health Sciences, Elizabeth City State University Campus of The University of North Carolina, Elizabeth City, NC 27909, USA; 3 Department of Cell Biology, Harvard University, Boston, MA-02115, USA

**Keywords:** Carbon Nano Tube, Triple Negative Breast Cancer, Epithelial Mesenchymal Transition, Spheroids, Nanotechnology, Theragnostic

## Abstract

Triple Negative Breast Cancer (TNBC) is a malignant cancer with a very high mortality rate around the world. African American(AA) women are 28% more likely to die from triple-negative breast cancer (TNBC) than white women with the same diagnosis. AA patients are also more likely to be diagnosed at a later stage of the disease and have the lowest survival rates for any stage of diagnosis; There are very few existing anti TNBC drugs with therapeutic efficacy hence newer anti TNBC drug design and investigation is needed. Carbon Nano Tubes(CNT) in recent years have shown effective anti-cancer properties in various types of cancers as reported in peer reviewed journals. Henceforth, we did an investigation to study the anticancer properties of a novel CNT in both *in vitro* and *in vivo* models of TNBC. We tested the CNT drug *in vitro* cytotoxicity studies on TNBC model MDA-MB-231 VIM RFP cell lines and Spheroid forming assays on the same cancer cells; we also did an *in vivo* study on TNBC model mice to study the therapeutic efficacy of this CNT drug in reducing the tumor load. Our initial studies showed increased cell death and reduction in spheroid numbers in the CNT treated cancer cells in comparison to control and a significant reduction in the tumor volume in the TNBC model mice than in untreated animals. Thus our initial studies have shown significant therapeutic efficacy of the novel CNT as an anti TNBC agent. Additional mechanistic studies need to be done to find out the cell death mechanisms, core canonical pathways involved, pharmacokinetic studies before translational research for this novel nanoparticle as a therapeutic agent from bench to bedside.

## INTRODUCTION

Carbon nanotubes (CNTs) have garnered significant interest in various fields due to their unique properties, researchers are exploring their potential applications in cancer treatment. Some potential ways in which carbon nanotubes are being investigated for cancer treatment include targeted drug delivery; CNTs can be functionalized to attach specific targeting molecules, such as antibodies or peptides, which can recognize and bind to cancer cells. This targeted approach enhances drug delivery to cancer cells while minimizing damage to healthy cells, CNTs can encapsulate and carry therapeutic drugs, genes, or other treatment agents. This can help improve the solubility and stability of certain drugs and enhance their delivery to cancer cells. CNTs exhibit strong absorption in the near-infrared (NIR) region, allowing them to convert light into heat. In photothermal therapy, CNTs can be selectively accumulated in tumors and then irradiated with NIR light. This results in localized heating, damaging cancer cells while sparing normal tissue [1–4]. CNTs can be used as contrast agents in various imaging techniques, such as magnetic resonance imaging (MRI) and photoacoustic imaging. This can aid in the early detection and monitoring of tumors. CNTs can enhance the effectiveness of radiotherapy cells, CNTs can be utilized for the development of biosensors to detect cancer biomarkers. This can contribute to early diagnosis and monitoring of cancer progression. When irradiated with X-rays, CNTs can produce reactive oxygen species, enhancing the damage to cancer cells [5–8]. Henceforth, in this study, we were interested to study the anticancer therapeutic properties of CNTs in a triple negative breast cancer cell line.

Triple-negative breast cancer (TNBC) is a subtype of breast cancer that lacks expression of estrogen receptor (ER), progesterone receptor (PR), and human epidermal growth factor receptor 2 (HER2). These receptors are proteins that can promote the growth of some types of breast cancer.

TNBC accounts for approximately 10–15% of all breast cancers, It is more common in younger women, African American women, and those with a BRCA1 mutation. TNBC tends to be more aggressive than other types of breast cancer, It may have a higher rate of recurrence and metastasis. TNBC lacks the typical receptors targeted by some breast cancer treatments (such as hormonal therapies and HER2-targeted therapies) hence treatment options are more limited [9–16].

Epithelial Mesenchymal Transition (EMT) is a highly dynamic process, by which epithelial cells can convert into a mesenchymal phenotype. However, it is also involved in tumor progression with metastatic expansion, and the generation of tumor cells with stem cell properties that play a major role in resistance to cancer treatment. There is a great demand for antineoplastic agents that could prevent EMT in cancer cells [17–19].

This study is reporting the results of an investigation on treating an EMT model of triple negative breast cancer cell lines with a novel CNT for therapeutic purposes and novel anti-cancer drug discovery.

## MATERIALS AND METHODS

### Cell Lines

MDA-MB-231 VIM RFP is a fibroblast-like reporter labeled cell line purchased from ATCC (USA) that was isolated from the pleural effusion of a 51-year-old, White, female with adenocarcinoma. This cell line can be used in drug development research. MDA-MB-231 VIM RFP reporter cell line provides a convenient and sensitive platform for research on the mechanisms of metastasis *in vitro* and the development of new anti-Epithelial Mesenchymal Transition drugs for metastatic breast cancer.

### CNT

Carboxylic acid functionalized double-wall carbon nanotubes, which were produced via the Catalytic Chemical Vapor Deposition (CCVD) process were purchased from the Nanocyl (NC3101, Belgium). This multi-walled carbon nanotube’s outer diameter and length were approximately 9.5 nm and 1.5 μm, respectively, resulting in a notably high aspect ratio. The nanotubes also exhibited COOH functionalization less than 8%.

### Cytotoxicity Assay

Cell Counting Kit-8 (CCK-8) purchased from Promega (USA) provides a more convenient and sensitive method for the research of cell number determination and cell proliferation to toxicity assay. The kit utilizes a highly water-soluble tetrazolium salt, WST-8, which produces a water-soluble formazan dye upon reduction in the presence of an electron mediator. The amount of the formazan generated by dehydrogenases is directly in proportion to the numbers of living cells. TNBC cells were exposed to CNT at different doses for 48 hours incubation and CCK8 analysis was done using a standard microplate reader.

### Spheroid Forming Assay

MDA-MB-231 breast cells were grown to confluence on a Corning Ultra-Low Attachment surface six well plate. The ultralow attachment surface is a hydrophilic, neutrally charged coating covalently bound to a polystyrene vessel surface. The hydrogel inhibits specific and nonspecific immobilization which forces cells into a suspended state and enabling 3D spheroid formation. CNT treated and untreated spheroid numbers were counted using a standard inverted microscope.

### Murine Tumor Model and Treatment Procedure

The female nude immunodeficient mice (NU/J), 6 weeks old, were obtained from Jackson Laboratory (Bar Harbor, ME). Mice were allowed to acclimatize for 1 week before implantation of tumor cells. Mice were housed, 5 per cage, in an isolation room (temperature controlled at 24°C, 12 h light/dark cycles) in the Old Dominion University Animal Research Facility. Fresh sterile cages, bedding, and water were provided weekly. All animal use and handling were approved by the Institutional Animal Care and Use Committee (IACUC).

Mice were injected subcutaneously with 5 × 105 human triple-negative breast cancer cells (TNBCs, MDA-MD-231- VIM-RFP) in 50 μL Dulbecco’s phosphate buffered saline (PBS) in the left flank. The size of primary tumor was assessed by calipers. Tumors were allowed to grow to a diameter of approximately 4–6 mm before treatment. Animals were divided into two groups (control, and MWCNT), 3 mice were in CNT group and Two in control group. Groups including MWCNT received a total volume of 200 μg in 50 ml of PBS with 25G needle intratumorally. Tumor growth was measured at 24 h post treatment and twice weekly using a digital caliper, and volumes (v) were calculated using the standard formula v = ab2π/6, where a is the longest diameter, and b is the next longest diameter perpendicular to a. Mice were euthanized at the end of the follow-up period or at specified time points described in experimental designs or when they met the criteria described at experimental endpoints in the approved IACUC protocol.

## STATISTICAL APPLICATION

All statistical analyses were performed using JMP^®^ 17.2.0 software by the SAS Institute (Cary, NC, USA). Drug treated and control comparisons were analyzed with one-way ANOVA, followed by Student’s t-test for pairs with ANOVA’s probability > F less than 0.05.

## RESULTS

**Figure 1:**
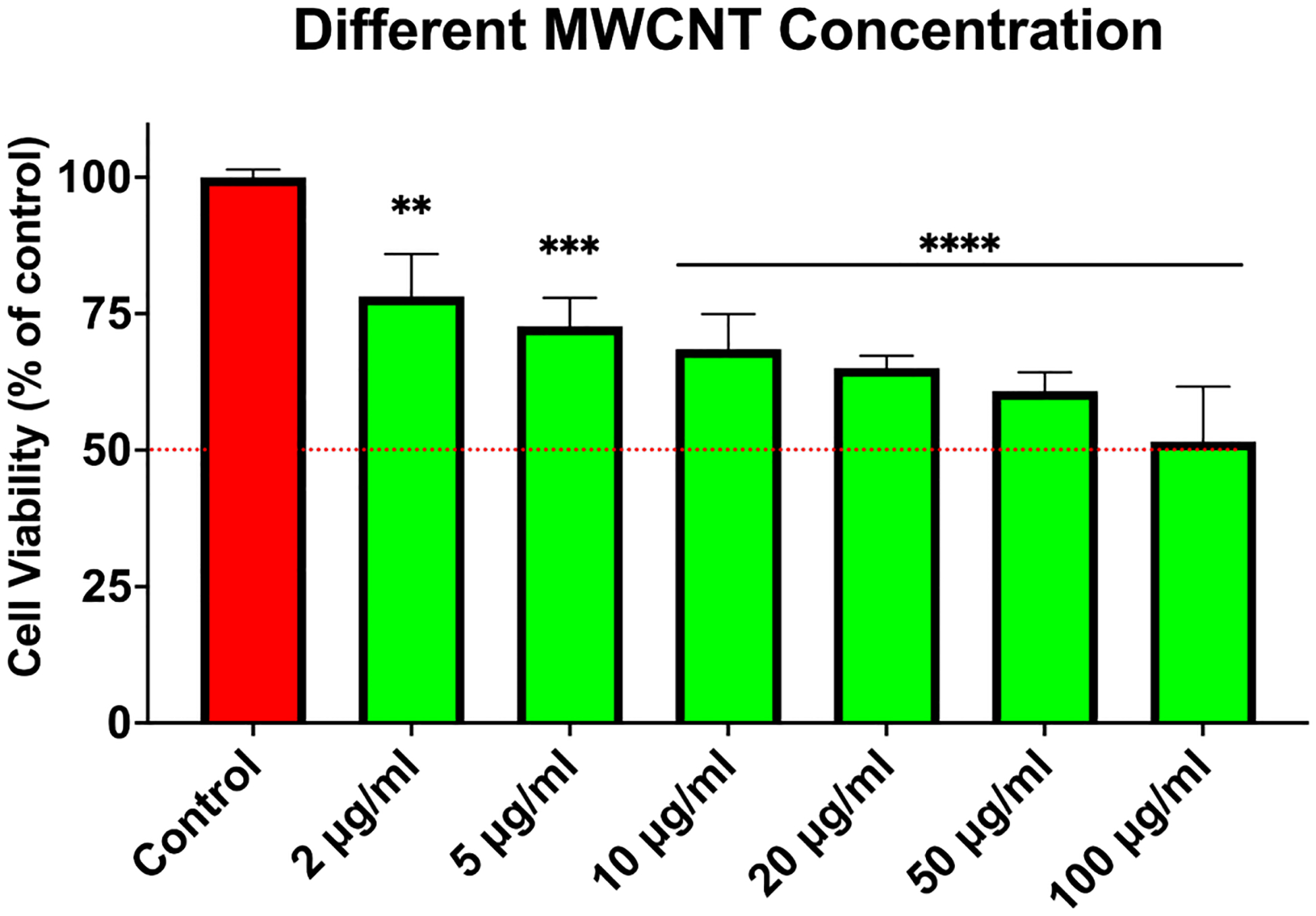
Cell viability study showing increased cytotoxicity of CNT with increased dose for 48 hours of incubation period and IC50 dose at 100ug/ml.

**Figure 2:**
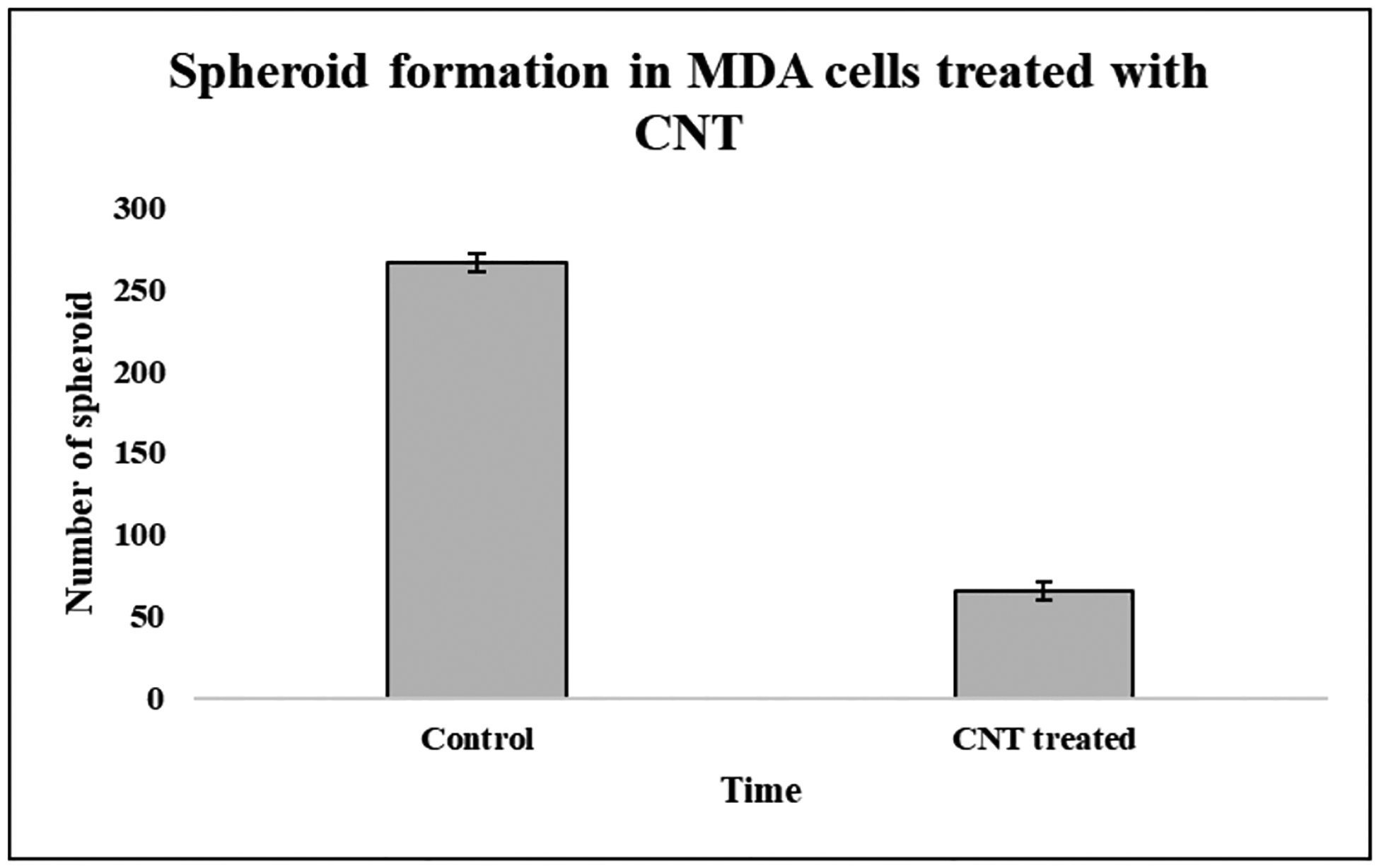
Spheroid forming assay showing decreased number of spheroids after CNT treatment when compared to untreated TNBC cells.

**Figure 3:**
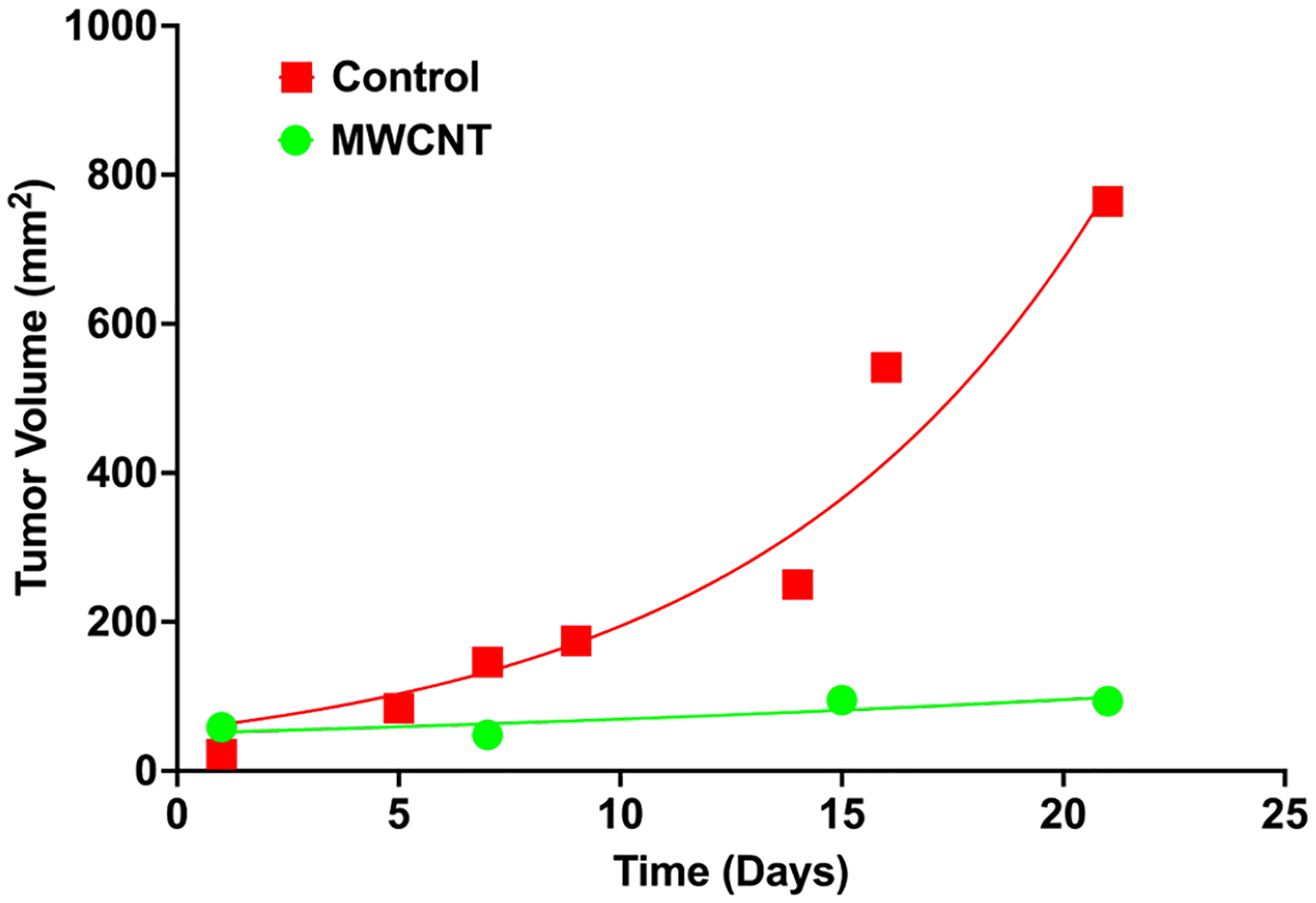
*In vivo* toxicity studies on TNBC model nude mice showed significant reduction of the tumor volume after CNT treatment in comparison to control animals.

CCK8 cytotoxicity assay showed the novel CNT successfully killing the TNBC cells with an IC50 dose of 100ug/ml (Fig. 1), Spheroid formation studies showed a 4 fold decreased number of spheroids in the CNT treated TNBC cells (Fig. 2); *In vivo* studies with TNBC model mice showed significant reduction in the tumor size in comparison to control untreated mice (Fig. 3).

## DISCUSSION

The overall five-year relative survival rate for TNBC is 77% as per current report of the American Cancer Society. Factors that can affect survival rates include age, overall health, and how well the cancer responds to treatment. TNBC accounts for 12% of breast cancers in the United States. About 80% of breast cancers, including TNBCs, are in people over 50 years old and African American ethnicity.

Triple-negative breast cancer (TNBC) doesn’t have estrogen or progesterone receptors and also makes too little or none of the HER2 protein. Because the cancer cells don’t have these proteins, hormone therapy and drugs that target HER2 are not helpful, so chemotherapy is the main systemic treatment option. TNBC tends to come back more frequently than other breast cancers after remission, henceforth, newer TNBC anticancer drugs research is very important and novel therapeutic drug candidates should be investigated.

During the multistep progression of carcinomas that are initially benign, epithelial cells acquire a few distinctly mesenchymal traits that confer them the ability to invade adjacent tissues, locally, and then disseminate to distant tissues. Much of this phenotypic progression towards increased invasiveness depends on the activation of EMT leading to drug resistance and cancer stem cell formation. There are very few anti-cancer agents till today that could successfully prevent EMT formation in the Tumor Micro Environment (TME), [20].

This study with novel carbon nanotubes as anti-cancer agent in treating an EMT model of TNBC cell line showed therapeutic efficacy in both *in vitro* and *in vivo* studies with a significant reduction in the TNBC tumor volume in nude cancer model mice experiments; this is a significant finding since there are very few existing anti TNBC drugs that could selectively kill these highly malignant cancer cells. Mechanistic studies including transcriptomic maps needs to be generated for additional information regarding the therapeutic properties of this novel CNT drug candidate as a new anti-cancer agent and its role in preventing Epithelial to Mesenchymal Transition and its role in the TME.

While the potential applications of carbon nanotubes in cancer treatment are promising, it’s important to note that research in this field is still in the preclinical and early clinical stages. Challenges such as biocompatibility, toxicity, and large-scale production need to be addressed before widespread clinical implementation. Regulatory considerations and long-term safety assessments are crucial for the development of CNT-based therapies [21–26]. Meng *et al*. showed an absence of systemic effects of CNTs by investigating the organ toxicity and immunological reactions induced following subcutaneous administration of 1 mg of carboxylated CNTs to BALB/c mice. Histological analysis of heart, liver, kidney, and spleen excised from the mice over a period of 2 to 90 days post MWNTs injection revealed normal histology with no apparent accumulation of CNTs [27].

## CONCLUSION

CNT could be an effective treatment option for TNBC both by itself or as an adjuvant to existing TNBC therapy, however, additional investigations will be required to take this drug from bench to bedside for clinical trials.
